# Characterization of Pathogenic Bacteria Isolated from Sudanese Banknotes and Determination of Their Resistance Profile

**DOI:** 10.1155/2018/4375164

**Published:** 2018-09-24

**Authors:** Noha Ahmed Abd Alfadil, Malik Suliman Mohamed, Manal M. Ali, El Amin Ibrahim El Nima

**Affiliations:** ^1^Department of Pharmaceutics, Faculty of Pharmacy, University of Al Neelain, Khartoum, Sudan; ^2^Department of Pharmaceutics, Faculty of Pharmacy, University of Khartoum, Khartoum, Sudan; ^3^Department of Pharmaceutical Microbiology, Faculty of Pharmacy, Omdurman Islamic University, Khartoum, Sudan

## Abstract

**Background:**

Banknotes are one of the most exchangeable items in communities and always subject to contamination by pathogenic bacteria and hence could serve as vehicle for transmission of infectious diseases. This study was conducted to assess the prevalence of contamination by pathogenic bacteria in Sudanese banknotes, determine the susceptibility of the isolated organisms towards commonly used antibiotics, and detect some antibiotic resistance genes.

**Methods:**

This study was carried out using 135 samples of Sudanese banknotes of five different denominations (2, 5, 10, 20, and 50 Sudanese pounds), which were collected randomly from hospitals, food sellers, and transporters in all three districts of Khartoum, Bahri, and Omdurman. Bacterial prevalence was determined using culture-based techniques, and their sensitivity patterns were determined using the Kirby–Bauer disk diffusion method. Genotypic identification was carried out using PCR and 16S rDNA sequencing. Antibiotic resistance genes of some isolates were detected using PCR technique.

**Results:**

All Sudanese banknotes were found to be contaminated with pathogenic bacteria. *Klebsiella pneumoniae* was found to be the most frequent isolate (23%), whereas *Bacillus mycoides* (15%) was the most abundant Gram-positive isolate. There was a significant relationship between the number of isolates and the banknote denomination with *p* value <0.05 (the lower denomination showed higher contamination level). Our study has isolated bacteria that are resistant to penicillins and cephalosporins. Multidrug-resistant strains harboring resistant genes (*mecA*, *bla*CTX-M, and *bla*TEM) were also detected.

**Conclusion:**

All studied Sudanese banknotes were contaminated with pathogenic bacteria, including multidrug-resistant strains, and may play a significant role in the transmission of bacterial infections.

## 1. Introduction

People in almost all communities exchange money on a daily basis, thus circulating banknotes might play a role as a potential vehicle for the transmission of bacterial infection and multidrug-resistant pathogens especially in immune-compromised people [1–8]. Banknotes may be contaminated during transaction, handling, storage, and upon contact with dirty surfaces. Unhygienic practices such as wetting fingers with saliva prior to money counting can introduce an array of bacteria to the notes [9, 10]. These routes of transmissions have a great impact on public health in developing countries like Sudan, where the frequency of bacterial infection has been increasing [11]. Although every location has specific endemic bacteria, members of *Enterobacteriaceae*, *Bacillus* spp., *Staphylococcus* spp., *Micrococcus* spp., and *Corynebacterium* spp. have been identified as common contaminants isolated from banknotes in different countries [1–3, 6, 12–21]. Moreover, bacteria belonging to the genera *Vibrio* and *Pseudomonas* have been isolated from banknotes in developing countries [2, 20]. Furthermore, there is accumulated data highlighting the contamination of banknotes by antibiotic-resistant bacteria [3, 13, 22–27]. The widespread occurrence of these bacterial species could lead to outbreaks of infections that might result in high morbidity and mortality with significant economic implications [28–31].

This study aimed at determining the prevalence of pathogenic bacteria in Sudanese banknotes, investigating their resistance profile, and characterizing some antibiotic resistance genes in bacterial chromosomes.

## 2. Methods

### 2.1. Sample Collection and Transport

Sample collection was done from November 2015 to March 2016. A total of 135 Sudanese banknotes were randomly collected from different sources: hospitals (*f* = 45), food sellers (*f* = 45), and transporters (*f* = 45). Five “mint” brand new notes were collected from the bank before being touched by bankers; these were used as controls. These new banknotes were included in the study to determine whether the banknotes are contaminated from their source or during handling in circulation. The banknotes studied were two, five, ten, twenty, and fifty Sudanese pounds (*f* = 27 for each one of them). Collected notes were categorized according to their physical status as clean (*f* = 36), dirty (*f* = 69), very dirty (*f* = 30), and mint (*f* = 5) and transported in sterile plastic Petri dishes to the microbiological laboratory for bacterial isolation, identification, and antibiotic sensitivity testing.

### 2.2. Isolation and Identification of Banknotes Contaminants

The banknotes were moistened with sterile distilled water, swabbed on both sides, and swab inoculated onto 5% blood agar plates, selective and differential media (MacConkey's agar, cetrimide agar, xylose lysine deoxycholate agar, and Mannitol salt agar). Plates were incubated aerobically at 37°C for 24 h and observed for bacterial growth. Pure cultures were prepared by streaking a small amount of cells from a distinct colony on a nutrient agar plate and incubated for 24 h at 37°C. Isolated colonies after incubation were pure cultures. Pure cultures were identified based on their colonial morphology, Gram reaction, and biochemical tests including indole production, citrate utilization, urease activity, growth on Kligler iron agar (KIA) fermentation of glucose, lactose, and maltose, motility, oxidase production, starch hydrolysis, catalase production, casein hydrolysis, and the MR-VP test, according to protocols previously described [32, 33].

### 2.3. Antibiotic Susceptibility

Antibiotic susceptibility was determined by Kirby–Bauer disc diffusion technique on Mueller-Hinton agar [32], using 18 antibiotic discs supplied by Hi-Media Laboratories, India. Antibiotics tested correspond to the drugs most commonly used in Khartoum State for the treatment of bacterial infections. The discs used were ampicillin (AMP) 10 *µ*g, amoxicillin (AMX) 25 *µ*g, amoxiclav (AMC) 30 *µ*g, cephalexin (CN) 30 *µ*g, cefuroxime (CXM) 30 *µ*g, ceftriaxone (CTR) 30 *µ*g, ceftazidime (CAZ) 30 *µ*g, gentamicin (GEN) 10 *µ*g, amikacin (AK) 30 *µ*g, co-trimoxazole (COT) 25 *µ*g, penicillin-G (P) (10IU), erythromycin (E) 5 *µ*g, azithromycin (AZM) 15 *µ*g, ciprofloxacin (CIP) 30 *µ*g, levofloxacin (LE) 5 *µ*g, nitrofurantoin (NIT) 200 *µ*g, chloramphenicol (C) 30 *µ*g, and meropenem (MEM) 10 *µ*g. Zone of inhibition size interpretative criteria was according to EUCAST standard (the European Committee on Antimicrobial Susceptibility) [34].

### 2.4. Chelex Method for Genomic DNA Extraction

Representative samples (61 cultures of 270 isolates) covering all species identified by culture-based techniques were selected for genotyping. Three colonies of each isolate were added to 300 *µ*l (1×) phosphate buffer solution and genomic DNA was extracted by vortexing the mixture for ten seconds and then centrifuged at 10,000 rpm for five minutes. The supernatant was discarded and the pellet resuspended in 200 *µ*l of 6% Chelex, swirled at moderate speed, and incubated at 56°C for thirty minutes. After incubation, it was boiled for fifteen minutes at 100°C, vortexed for ten seconds, and cooled to room temperature, and the mixture was centrifuged for one minute at 10,000 rpm. Finally, the supernatant was transferred into a clean 0.5 ml Eppendorf microfuge tube [35]. The dsDNA and protein concentrations (both in *µ*g/ml), ratio of dsDNA/protein, and purity (in percent) were quantified by GeneQuant (Biochrom Ltd, England) according to the manufacturer's instructions.

### 2.5. PCR Amplification and Detection of 16S rDNA and Resistance Genes

Genomic DNA of 61 cultures that exhibited marked resistance patterns toward beta-lactam antibiotics was selected to determine beta-lactamase-producing (BLP) bacterial strains. PCR amplifications of 16S rDNAs using universal primers (27F: 5′-AGAGTTTGATCCTGGCTCAG-3′, 1495R: 5′-CTACGGCTACCTTGTTACGA-3′) and three resistance genes (*bla*TEM, *bla*CTX-M, and *mecA*) were done using Maxime PCR PreMix kit (iNtRON Biotechnology). To amplify the genes, 13 *µ*l of distilled water and 1 *µ*l of forward and reverse primers were added to Eppendorf tube containing 15 *µ*l of PreMix (ITRON, Korea), and 5 *µ*l of DNA extract was added to each PreMix Eppendorf tubes. The PCR was carried out using conventional thermal cycler. Genomic DNA of 50 bacterial isolates was screened for the presence of *bla*CTX-M gene using known primers (F: 5′-TTTGCGATGCATACCAGTAA-3′ and R: 5′-CGATATCGTTGGTGCCATA-3′) [36] and *bla*TEM genes (F: 5′-ATGAGTATTCAACATTTCCG-3′ and R: 5′GTCACAGTTACCAATGCTTA-3′) [37], whereas DNA of 11 *Staphylococcus aureus* and coagulase-negative *Staphylococci* (CONs) isolates was screened for the presence of *mecA* genes using primers (*mecA* F: 5′-GTAGAAATGACTGAACGTCCGATGA-3′, *mecA* R: 5′-CCAATTCCACATTGTTTCGGTCTAA-3′) [38]. PCRs were carried out at starting denaturation temperature of 95°C for 5 min, and 35 cycles of denaturation at 95°C for 1 min, annealing at 58°C for *bla*TEM, at 60°C for *bla*CTX-M for 1 min, and at 50°C for *mecA* for 45 sec, and extension at 72°C for 1 min with final extension at 72°C for 10 min. The intended gene bands were confirmed on gel electrophoresis with the aid of UV transilluminator.

### 2.6. Statistical Analysis

Data were analyzed descriptively using SPSS 16.0 software. One-way analysis of variance (ANOVA) was used to determine the associations between variables where *P* ≤ 0.05 is statistically significant.

### 2.7. Sequencing of 16S rDNA and NCBI Accession Numbers

16S rDNA of representative samples of 14 clinically important species identified using culture-based technique was sent to Macrogen company (Seoul, Korea) for further DNA purification and standard sequencing, in order to verify the results of biochemical-based methods for bacterial identification at species level. The obtained sequences with different lengths (bp) were viewed and corrected manually using Finch TV program (http://www.geospiza.com/Products/finch tv.shtml) and compared with similar sequences in the GenBank using BLAST server (http://blast.ncbi.nlm.nih.gov/Blast.cgi) to determine the identity scores to similar sequences in the database. High-quality 16S rDNA sequences determined in this study were deposited in NCBI to get accession numbers.

## 3. Results

### 3.1. Types of Bacterial Contaminants in Banknotes

One hundred forty (135 samples and 5 controls) Sudanese banknotes were examined for bacterial contamination, and all samples were found to be contaminated with different species of bacteria. There was no bacterial growth detected on the control samples collected from Central Bank of Sudan where banknotes are printed (*N* = 5). Two hundred seventy organisms were recovered from the samples. Mixed contamination with both Gram-negative and Gram-positive bacteria observed in a high percentage 51.9% (70/135) of tested notes. Samples strictly contaminated with either Gram-negative or Gram-positive bacteria represented 23.7% (32/135) and 24.4% (33/135), respectively. One hundred fifty-six Gram-negative bacteria were isolated from Sudanese banknotes. *Klebsiella pneumoniae* was found to be the most frequent isolate (23%, *N* = 31/135), followed by *Pseudomonas stutzeri* (15%, *N* = 21/135), *Escherichia coli* (12%, *N* = 16/135), *Pseudomonas aeruginosa*, and *Citrobacter freundii* sharing the same percentage (9%, *N* = 12/135); *Acinetobacter baumannii* and *Salmonella paratyphi* A sharing the same percentages (7.4%, *N* = 10/135); *Proteus mirabilis* (4.4%, *N* = 6/135), *Alcaligenes faecalis* (3.7%, *N* = 5/135), *Enterobacter cloacae*, and *Serratia marcescens* sharing the same percentages (3%, *N* = 4/135); and *Shigella flexneri* (2.2%, *N* = 3/135). Nine different species of 114 Gram-positive bacterial contaminants were isolated from Sudanese banknotes, in which *Bacillus mycoides* were found to be the most abundant (15%, *N* = 21/135), followed by *Bacillus subtilis* (14.8%, *N* = 20/135), *Bacillus cereus* (12.5%, *N* = 17/135), *Micrococcus luteus* (10%, *N* = 14/135), *Staphylococcus haemolyticus* (8.8%, *N* = 12/135), *Staphylococcus aureus*, and *Staphylococcus saprophyticus* having the same percentage (8.1%, *N* = 11/135), *Bacillus horikoshii* (3.7%, *N* = 5/135) and *Staphylococcus epidermis* (2.2%, *N* = 3/135).

Out of 270 bacterial isolates obtained from banknotes, 61 were subjected to 16S rDNA amplification using universal primers. Fourteen of them were confirmed using 16S rDNA sequencing methods in order to verify the results of phenotyping for bacterial identification at species level. Nucleotide BLAST search gave 99-100% identity score to the sequences in the database. The comparison between the conventional identification and the 16S rDNA sequence identification showed same results for both methodologies the 14 bacterial isolates selected. These isolates have been registered in GenBank under accession numbers *(Klebsiella pneumoniae* (KY049979), *Klebsiella oxytoca* (KY031321), *Escherichia coli* (MG198700), *Pseudomonas aeruginosa* (MG198702), *Citrobacter freundii* (MG198698), *Acinetobacter baumannii* (KY114514), *Alcaligenes faecalis* (MG198707), *Enterobacter cloacae* (KY205640), *Bacillus horikoshii* (MG198701), *Staphylococcus aureus* (KY176381), *Staphylococcus haemolyticus* (MG198699), *Proteus mirabilis* (JF947362), *Bacillus cereus* (JX218990.1), and *Shigella flexneri* (KY199565)).

### 3.2. Relationship between Bacterial Contamination and Denomination of Banknotes

There was a significant relationship between the number of isolates and banknote denomination with *p* value < 0.05. The lower denomination 2 and 5 Sudanese Pounds showed higher contamination level with mixed species of bacteria (by 63% for both) than higher denomination 10, 20, and 50 Sudanese Pounds (by 37%, 51%, and 44.5%, respectively).

### 3.3. Relationship between Bacterial Contamination and Physical State of Banknotes

Out of 135 of collected notes, 69 (51%) were dirty, 36 (27%) were clean, and 30 (22%) were very dirty.

There was a no significant relationship between the number of isolates and the physical appearance of banknotes (*p* value <0.05). Contamination by mixed species of bacteria was observed with high percentages in clean, very dirty, and dirty banknotes by 58% (21/36), 50% (15/30), and 49% (34/69), respectively. The prevalence of Gram-negative bacteria was high in clean (27.8%) and dirty (30.4%) notes as opposed to the recovery in very dirty notes in which Gram-positive bacteria were more abundant (30%) compared to Gram-negative bacteria.

### 3.4. Relationship between Bacterial Contamination and Place of Banknote Collection

There was no significant relationship between the type of bacteria contaminating and source of banknotes (*p* > 0.05). Contamination by Gram-negative bacteria in Bahri (*N* = 45) was 33.3%. This was more than the 24.4% contamination by Gram-positive bacteria. However, notes from Omdurman and Khartoum (*N* = 45) had similar frequencies of occurrence of bacteria, in which Gram-positive bacteria in Khartoum (26.7%) and Omdurman (22.2%) were more prevalent than Gram-negative bacteria, 17.8% and 20.0%, respectively.

### 3.5. Antibiotic Resistance Testing: Correlation between Results of Phenotypic and Genotypic Testing

Co-trimoxazole, quinolones (ciprofloxacin and levofloxacin), and meropenem were the most active drugs as they were effective against all isolates (100%). All examined isolates recovered from Sudanese banknotes were resistant to ampicillin (100%). However, some strains of *Proteus mirabilis*, *Serratia marcescens*, *Citrobacter freundii*, and *K. oxytoca* were found to be sensitive to amoxicillin and co-amoxiclav. Out of 61 genomic DNA subjected to antibacterial resistance analysis, 24 harbored the resistance genes; *bla*CTX-M gene was detected in PCR products of *E. coli, K. pneumoniae, Enterobacter cloacae, Acinetobacter baumannii*, and *P. aeruginosa*. However, *bla*TEM gene was detected in one strain of *E. coli*. Isolates of *S. aureus* and *CONS* harboring *MecA* gene in their chromosomes were also detected as shown in Tables 1 and 2.

## 4. Discussion

In this study, it was found that all examined Sudanese banknotes (100% (*N* = 135)) were contaminated with different species of bacteria. The first occurrence of bacteria in banknotes was reported by Pope and co-workers in 2000 [4]. Successive studies conducted on Indian rupee [2, 25], Bangladeshi taka [8], Iraqi notes [19], and Ghanaian notes [5] reported that banknotes were contaminated (100%) with pathogenic bacteria, which were similar to our findings from Sudanese banknotes. The contamination of Saudi [3] and Nepali [12] paper notes with pathogenic bacteria was found to be 98% and 75%, respectively.

Gram-negative bacteria observed in our study belonged to *Enterobacteriaceae* and *Pseudomonas* spp. They were more prevalent than Gram-positive organisms. *K. pneumoniae* was the most frequent Gram-negative isolate, while *Bacillus* spp. was the most abundant Gram-positive isolate. Different species of bacteria isolated in this study were very similar to those isolated in previous studies [8, 18, 39, 40]; however, studies carried out in Saudi Arabia [3], Pakistan [26], Ghana [5], India [25], Iraq [19], and France [13] established Gram-positive bacteria as the major isolates from the contaminated banknotes.

This study sorted out the diversity of bacterial distribution in Khartoum, Bahri, and Omdurman. We found that bacterial contaminations were most abundant in Omdurman. Some species like *Acinetobacter baumannii* and *Alcaligenes faecalis* recovered from examined notes were not reported among Sudanese notes contaminants in the previous study which was conducted in the same study sites [18]. These differences in bacterial patterns may be attributed to regional variation of bacterial profiles and habits of the local people [12, 27]. Temporal variation in profiles should be another explanation for the variation observed. Furthermore, presence of the genera of coliform bacilli such as *Escherichia, Klebsiella,* and *Salmonella* is suggestive of significant fecal contamination of banknotes that may reflect the poor local sanitation and personal hygiene. They might also signify a potential minefield for nosocomial infections.

Detection of *Bacillus* spp. in this study agreed with findings from Ghana and India [10, 21]. The ubiquitous nature of the *Bacillus* spp*.,* greater colonization ability, and the ability of its spores to resist environmental changes might help it to inhabit the banknotes.

The extent of bacterial contamination was found to be related to banknote denomination (*p* < 0.05), in which the lower denominations show higher levels of contamination especially by mixed Gram-positive and Gram-negative bacteria. In this context, these findings are consistent with recent reports [2, 8, 13, 17–20, 25, 26, 39]. Lower denominations have higher circulation rate, therefore increasing the possibility of mishandling compared with higher denomination.

It is worth noting that bacterial growth was not detected in 5 samples of mint “newly printed” banknotes. The lack of growth in these notes might be attributed to the fact that they had not been in circulation that exposed them to usage and handling. However, some researchers believed that uncirculated notes are contaminated with fastidious organisms and the media or culture conditions employed were inappropriate for their isolation [13].

Bacteria isolated in this project were subjected to conventional Kirby–Bauer disk diffusion technique to test their sensitivity patterns towards some antimicrobials. All bacteria recovered from Sudanese banknotes were resistant to penicillins except some strains of *Proteus mirabilis, Serratia marcescens*, and *K. oxytoca.* Furthermore, some strains of *K. oxytoca, Serratia marcescens*, and coagulase negative *Staphylococci* were found to be resistant to cephalosporins. In this study, notable multidrug-resistant *P. aeruginosa*, *Escherichia coli*, and *Klebsiella* spp. were isolated. Moreover, *Staphylococcus aureus* showed significant resistance to macrolides (erythromycin and azithromycin)*. mecA* was detected in all isolates of *S. aureus* and some strains of CONs isolates that exhibited resistance to penicillins, as shown in Tables 1 and 2.

It is known that infection by multidrug-resistant bacteria limit therapeutic options and subsequently facilitate the dissemination of these strains. The sensitivity patterns of isolates to antibiotics are harmonious with the patterns reported by Akoachere et al. [13], except co-trimoxazole which was inactive against their isolates. The resistance of isolates to penicillins was comparable to that reported by Ali et al. [26]. This can be explained by the regional variation of organisms and is directly proportional to the use and misuse of antibiotics in the community.

Increasing resistance to penicillins and third-generation cephalosporins by high prevalence of ESBL among members of *Enterobacteriaceae* has become a serious cause of nosocomial infections leading to treatment failure and consequent escalation in costs of hospitalization. Resistance to penicillins and cephalosporins was also confirmed genotypically by detecting the presence of *bla*TEM- and *bla*CTX-M-resistant genes with PCR. *bla*CTX-M gene was detected in the PCR products when *E. coli*, *K. pneumoniae*, *Enterobacter cloacae*, *Acinetobacter baumannii*, and *P. aeruginosa* chromosomes used as PCR templates, but was found to be absent in other bacteria, that might contradict their disk diffusion results.

Despite some isolates resistance to some beta-lactams, their relevant resistance genes (*bla*TEM) were not detected in this study, except in one strain of *E. coli*. Primer dimers or presence of ESBL genes in plasmid may explain the absence of *bla*TEM-1 in the PCR products. In addition, improper storage of primers and probes may cause them to degrade and lose specificity, which in turn affects the reaction efficiency [28]. It is assumed that if primers were relevant and genes were present in chromosomal material, the difference that might be observed in detection of ESBL-positive isolates by genotypic and phenotypic methods may be justified by the lower sensitivity of phenotypic methods and the influence of environmental factors on the incidence of resistance [37].

The detection of bacterial contamination in banknotes from all sources is a great concern and indicates that contaminated banknotes could pose a health threat particularly to immunocompromised individuals in hospitals or community level. Strategies to reduce the contamination of banknotes are recommended, and such strategies could include the use of plastic banknotes, which can be washed easily, using electronic ATM cards, raising the general awareness of people about the proper handling of banknotes as well as regular withdrawal of damaged notes by federal authorities. Finally, periodical screening for the patterns of antibiotics resistance in environmental bacteria using large sample size is highly recommended.

## 5. Conclusion

Sudanese banknotes are contaminated with pathogenic and potentially pathogenic bacteria. This contamination may play a significant role in the transmission of infections in Sudan. Also, this study revealed that multidrug-resistant strains of different isolates were prevalent in some Sudanese banknotes.

## Figures and Tables

**Table 1:** Resistance profile and resistance genes (*bla*TEM, *bla*CTX-M, or *mecA*) detected in pathogenic bacteria isolated from banknotes (*N* = 61).

Isolates	“Resistance profile”	“Resistance genes”
*Gram-negative isolates* (*N* *=* 49)		
*E. coli* (5 isolates)	(AMP), (AMX), (AMC), (CN), CXM), (CTR), (CAZ), and (NIT)	*bla*TEM and *bla*CTX-M (1\5), (5\5)
*Proteus mirabilis* (1 isolate)	(AMP), (AMX), (AMC), (CN), (CXM), (CTR), and (CAZ)	-(1\1)
*Serratia marcescens* (4 isolates)	(AMP), (AMX), (AMC), (CN), (CXM), (CTR), (CAZ)	-(4\4)
*P. aeruginosa* (5 isolates)	(GEN) and (AK)	*bla*CTX-M (1\5)
*P. stutzeri* (14 isolates)	—	-(14\14)
*K. oxytoca* (1 isolate)	(AMP), (AMX), (AMC), (C), and (AK)	-(1\1)
*Acinetobacter baumannii* (3 isolates)	—	*bla*CTX-M (2\3)
*Salmonella paratyphi* A (2 isolates)	(AMP), (AMX), (AMC), (CN), and (NIT)	-(2\2)
*K. pneumoniae* (6 isolates)	(AMP), (AMX), (AMC), (CTR), and (AK)	*bla*CTX-M (5\6)
*Enterobacter cloacae* (2 isolates)	(AMP), (AMX), (AMC), (CXM), and (CTR)	*bla*CTX-M (2\2)
*Shigella flexneri* (1 isolate)	(AMP), (AMX), (AMC), and (NIT)	-(1\1)
*Alcaligenes faecalis* (3 isolates)	(AMP), (AMX), (CXM), and (Ak)	-(3\3)
*Citrobacter freundii* (2 isolates)	(AMP), (AMX), (AK), and (NIT)	-(2\2)

*Gram-positive isolates* (*N* *=* 12)		
*S. aureus* (6 isolates)	(AMP), (P), (AMX), (AMC), (CN), (CXM), (AZM), (E), and (C)	*mecA* (6\6)
*CONS* (6 isolates)	(AMP), (P), (AMX), and (CXM)	*mecA* (2\6)

**Table 2:** Percentage resistance to each relevant antibiotic among all isolated Gram-negative and Gram-positive bacteria (*N* = 61).

Antibiotics	Percentage resistance
(1) Ampicillin (AMP) 10 *µ*g	(61) 100%
(2) Amoxicillin (AMX) 25 *µ*g	(39) 64%
(3) Amoxiclav (AMC) 30 *µ*g	(27) 44%
(4) Cefuroxime (CXM) 30 *µ*g	(18) 30%
(5) Cephalexin (CN) 30 *µ*g	(17) 28%
(6) Nitrofurantoin (NIT) 200 *µ*g	(8) 13%
(7) Ceftriaxone (CTR) 30 *µ*g	(8) 13%
(8) Ceftazidime (CAZ) 30 *µ*g	(6) 10%
(9) Amikacin (AK) 30 *µ*g	(6) 10%
(10) Gentamicin (GEN) 10 *µ*g	(2) 3%
(11) Chloramphenicol (C) 30 *µ*g	(1) 2%
(12) Ciprofloxacin (CIP) 30 *µ*g	(0) 0%
(13) Levofloxacin (LE) 5 *µ*g	(0) 0%
(14) Co-trimoxazole (COT) 25 *µ*g	(0) 0%
(15) Meropenem (MEM) 10 *µ*g	(0) 0%
(16) Penicillin-G (P) (10IU)	12^G^ (100%)
(17) Erythromycin (E) 5 *µ*g	5^G^ (42%)
(18) Azithromycin (AZM) 15 *µ*g	4^G^ (33%)

G: percentage resistance of examined Gram-positive bacteria isolates (*N* = 12).

## Data Availability

“Sequences data” that support the findings of this study have been deposited in “GenBank” under accession numbers (*Klebsiella pneumoniae* (KY049979), *Klebsiella oxytoca* (KY031321), *Escherichia coli* (MG198700), *Pseudomonas aeruginosa* (MG198702), *Citrobacter freundii* (MG198698), *Acinetobacter baumannii* (KY114514), Alcaligenes *faecalis* (MG198707), *Enterobacter cloacae* (KY205640), *Bacillus horikoshii* (MG198701), *Staphylococcus aureus* (KY176381), *Staphylococcus haemolyticus* (MG198699), *Proteus mirabilis* (JF947362), *Bacillus cereus* (JX218990.1), and *Shigella flexneri* (KY199565)).
